# Observation of Coalescence Process of Silver Nanospheres During Shape Transformation to Nanoprisms

**DOI:** 10.1007/s11671-010-9808-6

**Published:** 2010-09-28

**Authors:** Pyng Yu, Jane Huang, Jau Tang

**Affiliations:** 1 Research Center for Applied Sciences, Academia Sinica, Taipei, Taiwan

**Keywords:** Silver nanoprisms, Self-assembly, Cluster fusion, Coalescence, Recrystallization

## Abstract

In this report, we observed the growth mechanism and the shape transformation from spherical nanoparticles (diameter ~6 nm) to triangular nanoprisms (bisector length ~100 nm). We used a simple direct chemical reduction method and provided evidences for the growth of silver nanoprisms via a coalescence process. Unlike previous reports, our method does not rely upon light, heat, or strong oxidant for the shape transformation. This transformation could be launched by fine-tuning the pH value of the silver colloidal solution. Based on our extensive examination using transmission electron microscopy, we propose a non-point initiated growth mechanism, which is a combination of coalescence and dissolution–recrystallization process during the growth of silver nanoprisms.

## Introduction

Metal nanoparticles, due to collective electrons excitation known as surface plasmon (SP), offer a wide range of applications from tumor therapy, catalysis to enhanced solar cells [1,2]. These particles could provide strong localized EM fields at a nanoscale. Chemists made great stride in fine-tuning the properties of metal nanocrystals by controlling their sizes, shapes, compositions and structures. Compared with the conventional nanospheroids [3], nanoprisms exhibit a large red-shift of the surface plasmon resonance. Unlike nanospheroids, due to the large curvature of the tips on a nanoprism, an induced local electric field could be 3,500 times stronger in surface enhancing Raman scattering (SERS) than the incident electromagnetic field [4]. Several reports illustrated that an increase in thickness of nanoprisms results in a spectra shift about 10 times larger than the case in which the bisector length of a nanoprism is elongated [5,6]. Silver nanoprisms have high local refractive index in the NIR wavelength region, and they potentials in biomedical applications [7]. Recently, by coupling Ag nanoprisms to CdSe/ZnS core–shell quantum dots (QDs), the universal blinking could be suppressed and the fluorescence yield could be enhanced. Also, radiative decay rate would increase via tuning the distance between QDs and Ag nanoprisms. These improved characteristics could make the proposed scheme a better alternative for a high performance single-photon source [8,9].

The synthesis method of silver nanoprisms can be generally classified into three main categories: photochemistry [5,10], thermal method [11,12], and direct chemical reduction [6,13]. For this unique triangular shape, its growth mechanism has been studied by many groups, yet the details remain a mystery. Some hypotheses were proposed to explain the formation of the silver nanoprisms such as crystal-twinning theory [14,15], trimeric clusters model [16], and face-selective ligand passivation [17]. The crystal-twinning theory was first proposed by Berriman and Herz to specify the plate-like AgBr crystal in 1957. The generation of {111} twin planes and stacking faults are key factors influencing particle shape. It is well known that the facets comprising silver crystals have different surface free energies: *σ*_111_ <*σ*_100_ <*σ*_110_. Therefore, particle growth is accelerated parallel to the twin planes extending the lowest energy crystal facet {111}. Furthermore, in the study of Xia et al. [16], the silver trimeric clusters serve as the nucleation sites and they grow into prisms as more silver atoms gathered together.

Large triangular silver nanocrystals formed by mild annealing of silver nanoparticles on the carbon substrate were observed by Courty et al. [18] They first pointed out that coalescence and recrystallization took place on the substrate. Recently, Zeng et al. observed the growth trajectories of individual face cubic center (f.c.c.) platinum nanocrystal in solution by using a liquid cell that operates inside a transmission electronic microscope (TEM). They also demonstrated that both the coalescence and growth mechanisms might coexist simultaneously [19]. The coalescence in f.c.c. is also observed in some other experiments [20,21]. We reported here for the first time the observation of the formation of the silver nanoprisms in solution involving coalescence processes, very different from the point initiated growth mechanism.

## Experimental Section

In this study, we synthesized silver nanoprisms using a modified wet chemistry method developed by Mirkin et al., without the presence of strong oxidant H_2_O_2 _[6]. The procedure includes the preparation of spherical Ag nanoparticles, followed by transformation of the Ag nanospheres into triangular nanoplates in 24 h at room temperature and without light illumination. In a typical experiment, all the apparatus were washed by aqua regia and rinsed with acetone and DI water before use. A volume of 112 mL aqueous solution containing silver nitrate (AgNO_3_), 8.9 × 10^-5^ M, trisodium citrate (Na_3_C_6_H_5_O_7_), 1.6 × 10^-3^ M, and poly(vinylpyrrolidone) (PVP), 3.75 × 10^-5^ M were mixed. Then, 1,100 μL of 0.1 M sodium borohydride (NaBH_4_) was injected into the mixture. The effect of NaBH_4_ was studied by varying its amounts from 800 to 1,300 μL. Either sodium hydroxide (NaOH) or nitric acid (HNO_3_) was added to the initial nanoparticles solutions (20 mL) in order to achieve pH control. The images of the synthesized nanoparticles were taken by a transmission electron microscope (JEOL, JEM-1200EX II) and high-resolution transmission electron microscope (JEOL, JEM-2010F) operating at 80 and 200 kV, respectively. All UV–visible absorption spectra of the silver solutions were measured by a spectrophotometer (JASCO V-570) with a light path of 10 mm.

## Results and Discussion

**Figure 1:**
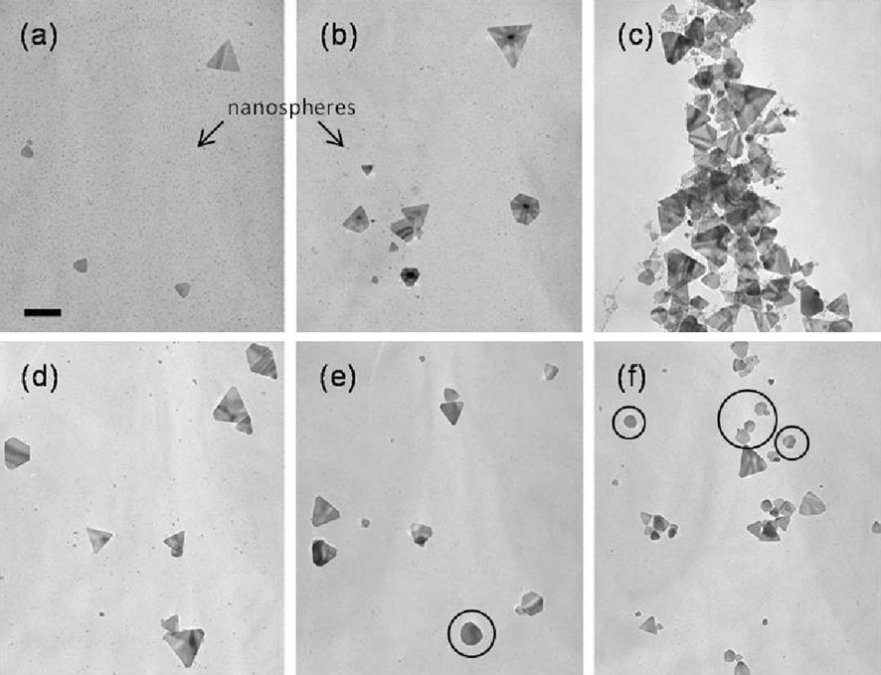
**TEM images of the silver nanoparticles prepared with different amounts of NaBH_4_**: **a** 800 μL, **b** 900 μL, **c** 1,000 μL, **d** 1,100 μL, **e** 1,200 μL, and **f** 1,300 μL. *Scale bar* is 100 nm.

**Figure 2:**
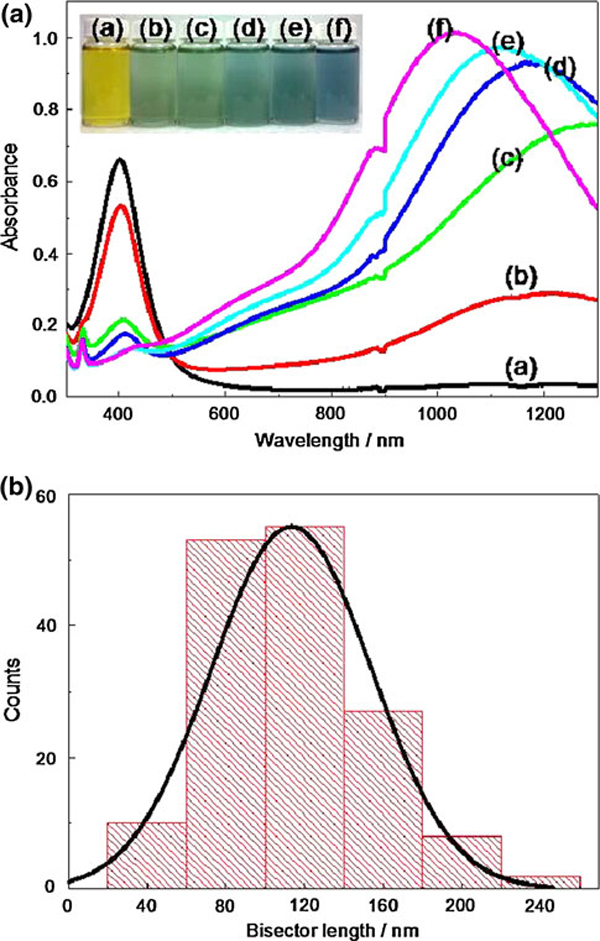
**a UV–Vis–NIR spectra of the silver nanoparticles prepared with different amounts of NaBH_4_**: *a* 800 μL (pH 9.28), *b* 900 μL (pH 9.34), *c* 1,000 μL (pH 9.36), *d* 1,100 μL (pH 9.45), *e* 1,200 μL (pH 9.46), and *f* 1,300 μL (pH 9.48). The *inset* shows photographs from *left* to *right* corresponding to concentration of NaBH_4_ low to high. **b** The histogram of the bisector length for the silver nanoprisms prepared by NaBH_4_ 1,100 μL, the bisector length is 113.4 ± 40.8 nm.

Figure 1 shows the transmission electron microscope (TEM) images of silver nanoparticles prepared with different amounts of NaBH_4_ while all other parameters remained constant. An extremely high mole ratio was used in this experiment, where the ratio of NaBH_4_ to AgNO_3_ is above 8. According to Yang et al. [22], the precursor (Ag ions) can be totally consumed when the mole ratio is 5. These prepared spherical Ag nanoparticles would transform into triangular nanoplates within 24 h at room temperature in dark. As the reaction proceeds, the intensity of the peak at 410 nm decreased and a new peak at a longer wavelength appeared in the NIR–Vis–UV absorption spectrum. This red-shift in energy implies the formation of a triangular structure, which is ascribed to the in-plane dipole resonance mode of silver nanoprisms as shown in Figure 2a. The bisector length of the silver nanoprism prepared with 1,100 μL NaBH_4_ was found to be 113.4 ± 40.8 nm, as shown in Figure 2b for the histogram. In this study, the out-of plane dipole mode did not show in the spectra, suggesting an inhomogeneous mixture of anisotropic nanoparticles in the solution. From TEM images and absorption spectrum, three features were observed. First, as the concentration of reducing agent (NaBH_4_) increases, the ratio of nanoprism/nanosphere would also increase. Secondly, the size of the formed silver nanoprisms is almost identical regardless the concentration of NaBH_4_. Finally, the out-off plane quadruple resonance mode is fixed at the same wavelength (331 nm), which implies that the concentration of the reducing agent would not affect the thickness of nanoprism. Consequently, the extreme excess amount of reducing agent is the crucial factor for the transformation.

**Figure 3:**
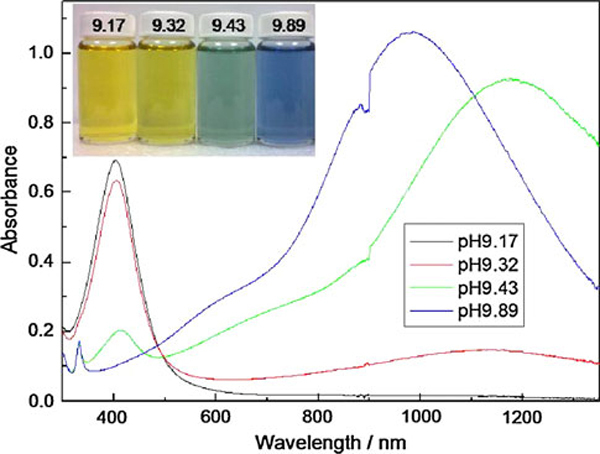
**UV–Vis–NIR spectra of the silver nanoparticles prepared with different pH value**: **a** pH 9.17, **b** pH 9.32, **c** pH 9.43, and **d** pH 9.89. The *inset* shows photographs from *left* to *right* corresponding to pH high to low.

When the amount of NaBH_4_ is increased above 1,100 μL, the tips of nanoprism would become more rounded and turn into a disk-like nanoplate, which is marked with circles in Figure 1e, f. As nanoprism transform into disk-like nanoplate, blue-shift in the in-plane dipole mode was observed [23]. To determine the effect of reducing agent concentration in shape transformation, the pH value of each as-synthesis nanoparticles was measured. The pH ranged from 9.28 to 9.48 and the pH increases as the concentration of reducing agent increases. The pH value corresponding to the amount of NaBH_4_ is listed in Figure 2a. To evaluate the effect of pH on shape transformation from spheres to nanoprisms, NaOH and HNO_3_ were added to the system to probe the particle dependence on pH. The pH values ranged from 9.17, upon the addition of HNO_3_, to 9.89, with the addition of NaOH. Even though the change in pH was relatively small, very large differences in NIR–Vis–UV absorption spectrum were observed as shown in Figure 3. At a relatively acidic condition (pH 9.17), the transformation does not proceed and there is no evidence of prism formation. At the other end of the pH, the in-plane dipole resonance peak at long wavelength was obtained. It is noticed that the blue-shift was observed at higher pH, which is consistent with NaBH_4_ variation data.

**Figure 4:**
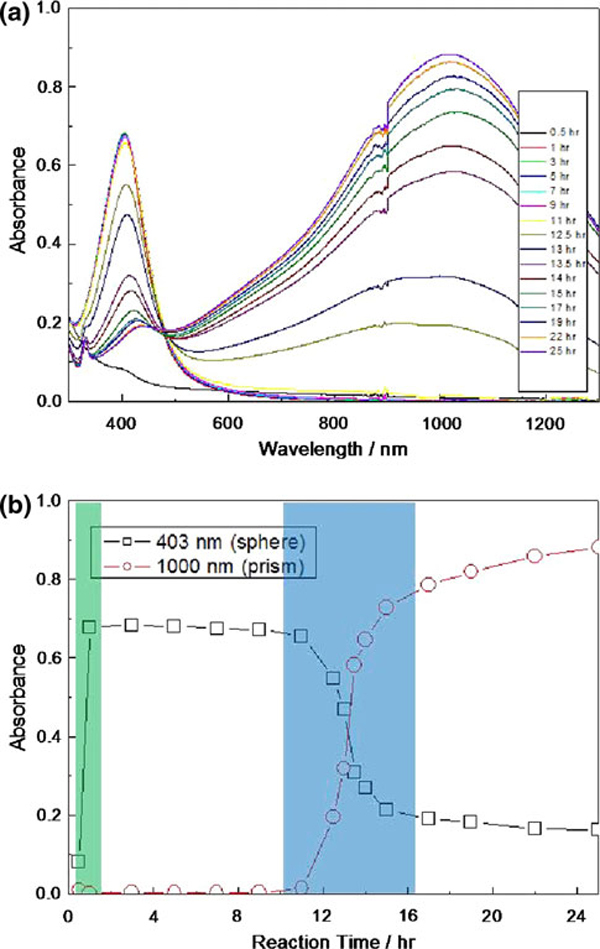
**a Time resolved extinction of the formation of the silver nanoprism via coalescence processes**. **b** Change in the absorbance at 403 nm (dipole band of the silver nanospheres) and 1,000 nm (in-plane dipole band of the silver nanoprisms) as a function of reaction time.

The synthesis was also carried out at different temperatures in the dark. We noticed that the transformation occurred even at the temperature down to 4°C in 2 weeks and the transformation occurred at room temperature in 24 h. Here, rising the reaction temperature would also increase the transformation rate from nanospheres into nanoprisms. However, as temperature increased to 37°C, smaller nanoprisms and irregular-shaped particles were formed within 24 h. In addition, there were only spherical silver nanoparticles obtained in the absence of citrate. Therefore, the citrate is not only capping molecules but also a reductant for the reaction over several hours in this synthesis. These results suggest that the strong oxidant, H_2_O_2_, addition, light, and heat are not required for the shape transformations. Figure 4a shows the time evolution of UV–Vis–NIR extinction spectra of the synthesized colloid (spherical nanoparticles) and nanoprisms. In the first hour, silver nanoclusters were formed (light yellow) then turned into nanoparticles immediately (yellow). The shape transformation of nanoprisms would occur automatically within 24 h (blue). These two transformation periods were marked in green and blue in Figure 4b, respectively. It is worth noticing that the peak position at the longer wavelength is fixed and only the intensity increased with time. Since the in-plane dipole resonance peak is very sensitive to the size and the aspect ratio, the result suggested that only the amount varies during the transformation while the size of the triangular structure remained constant.

**Figure 5:**
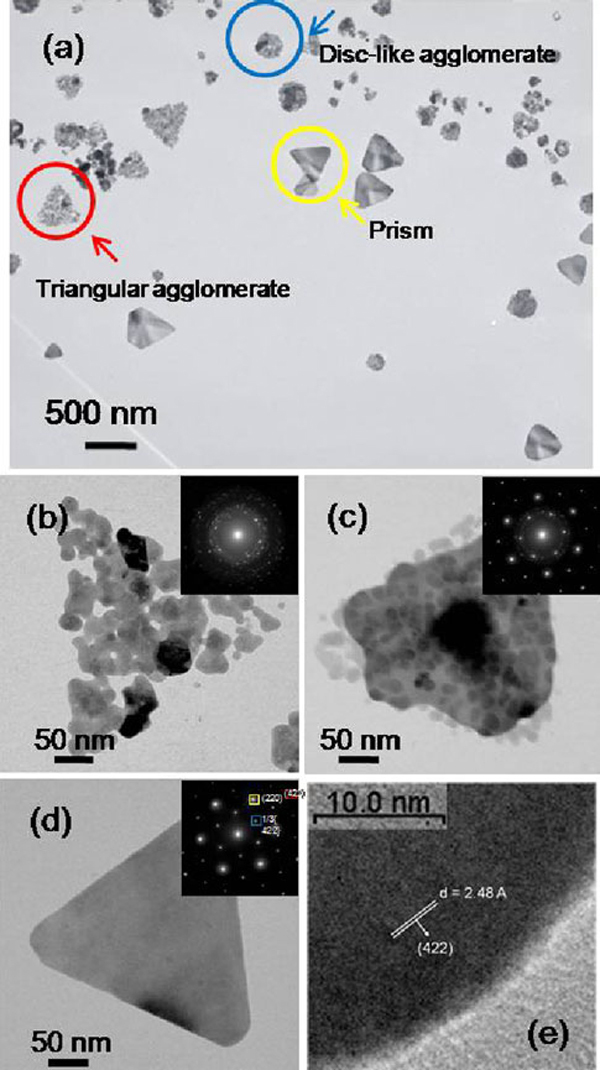
**a High-resolution TEM images of the samples prepared by NaBH_4_ = 1,100 μL and PVP = 6 mL**. The sample was pre-adsorbed on the amino-terminated silica film–coated copper grids to increase the adhesion of anionic nanoprisms. The subplots **b**–**d** show the individual nanostructure with a triangular shape. The *insets* show the electron diffraction pattern taken from the individual nanostructure. **e** HRTEM image of a silver nanoprism by directing the electron beam perpendicular to the flat face.

In this study, the "triangular and disk-like agglomerates" were observed as marked in Figure 5a. The surface of silicon film–coated copper grid was modified by the amino-terminate group (APTMS) to immobilize silver nanoparticle, which would reduce the self-assemble of silver nanoparticles during the evaporation. Then, the grid was immersed into the silver colloidal solution. With close examination of the TEM image, the particle–particle adhesion and coalescence by sintering would have a decreased free energy of the system due to the reduction of the interfacial area. Figure 5b–e are the high-resolution TEM images of some nanostructures with triangular shapes. The insets show the electron diffraction pattern taken from an individual nanoparticle indicating a polycrystalline, composite of a single crystal and a polycrystalline structure, and a single crystal, respectively. Figure 5c shows a layer of fused particles with a plate standing on top of the spherical particles. The presence of fused and unfused particles indicates that the nanoprisms were formed not through point initiated vectorial growth but, rather, by the recrystallization of multiple nanospheres in a triangular aggregate then fused gradually into one crystal. From the diffraction pattern, the fused particle was found to be single crystalline structure, which is identical to a silver nanoprism. Polycrystalline structures also exist in the diffraction pattern, which were contributed by the spherical particles or the unfused particles. Therefore, it should be considered as the intermediate structure of the transformation process. By analyzing the electron diffraction patterns, three sets of spots could be identified based on the d-spacing, i.e., the set with a spacing of 1.44 and 0.83 Å could be indexed to the {220} and {422} reflection of f.c.c. silver. Therefore, the top crystal face of the nanoprisms must be {111}. In addition, a set with a spacing 2.48 Å could be indexed to the 1/3{422} reflection of a hexagonal close-packed (h.c.p.) structure due to the atomically flat f.c.c. crystal surface of {111} as shown in Figure 5e. The silver nanoprism was found to be almost like a single crystal with {111} twin planes. These results were consistent with the previous observation of silver [24] and gold nanoprisms [25].

**Scheme 1:**
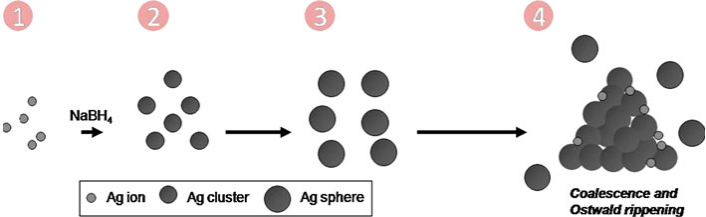
Schematic illustration of the formation of the silver nanoprism via coalescence/dissolution–recrystallization processes. *1* Ag ions; *2* Ag cluster was formed by reducing Ag ion; *3* There is an initial burst of production of small nanospheres in the presence of sufficient cluster concentration; *4* Ag nanoparticles possess lower reduction potential dissolved and others aggregated into a *triangular shape* simultaneously, then recrystallize to form a triangular plate. The length of *arrow* represents the consumed reaction time.

Scheme 1 shows a schematic diagram outlining a plausible mechanism, the details deserve future investigation. The silver clusters were formed by reducing AgNO_3_ with NaBH_4_:

(1) $${\text{BH}}_{4}^{-}+8{\text{Ag}}^{+}+8{\text{OH}}^{-}\to {\text{BO}}_{2}^{-}+6{\text{H}}_{2}\text{O}+8\text{Ag}$$

In the presence of sufficient concentration of clusters, there is an initial burst of nucleation of small nanospheres. Under this growth condition, the silver nanoparticles would comprise single-crystal structure and multi-twinned structure [26]. In this experiment, no additional Ag^+^ source was added. We also verified that if the solution was saturated by nitrogen to exclude the effects from the presence of oxygen, then no nanoprisms would form. Therefore, oxygen can chemically convert silver particles into Ag^+^ source.

(2) $$\text{Ag}+1/2{\text{O}}_{2}+{\text{H}}_{2}\text{O}\to {\text{Ag}}^{+}+2{\text{OH}}^{-}$$

In other words, the SPR of silver spherical nanoparticles exhibited a broad full-width at half-maximum (FWHM) of ~120 nm at an absorption of 403 nm as shown in Figure 4a. And the diameter of spherical nanoparticles ranged from 1 to 6 nm. According to the prior study [27], the smaller silver nanoparticles are more vulnerable to oxidation than larger ones. Also, the polycrystalline nanoparticles are more easily oxidized than single crystalline ones due to a lower reduction potential. For instance, the reduction potential of a 10 nm Ag nanoparticle is +769.0 mV and dramatically decreases to +490.4 mV when the diameter is at 1 nm.^1^ Since the smaller nanoparticles possess lower reduction potential and higher surface energy, they will dissolve in the solution, which contains oxygen, during the reaction.

At the same time, nanoparticles could also adhere and coalesce by the sinter process, leading to a decreased free energy of the system due to reduction of interfacial area. Then, a plate-like structure forms by extending the lowest surface energy crystal facet, {111}. Furthermore, the ions from dissolved particles would contribute to the growth of the prisms. Therefore, the formation of the silver nanoprisms could involve both coalescence and dissolution-recrystallization processes. Recently, Grouchko et al. reported the coalescence process that was induced by the self-assembly of silver nanoparticles to form 3D dendrites [20], which occurred during the evaporation of silver nanoparticles dispersion. They demonstrated that the f.c.c. structure particle could convert to h.c.p. structure via coalescence process by using in situ high-resolution TEM. The reorganization of nanoparticles into other nanostructures could occur in metal nanoparticles and also in semiconductors [28-32]. Mirkin et al. reported a bimodal distribution in the photochemical preparatory procedure for nanoprisms. In their study, since the large prisms (150 ± 16 nm) have an edge length approximately twice of the small prism (70 ± 12 nm), they proposed the formation through cluster fusion processes, where four small prisms could fuse and form one large prism [24]. Similarly, silver nanobelts have been prepared through thermal transformation from silver nanoprisms [11]. Therefore, both spherical and triangular nanoparticles can serve as building blocks in the fusion process. Our proposed mechanism differs from others who suggested that the formation of nanoprism is caused by point initiated growth [14-17].

## Conclusions

In summary, we used a direct chemical reduction method to study the growth mechanism of silver nanoprisms. We offered evidences to elucidate their growth mechanism and shape transformation. The extreme excess amount of reducing agent is the crucial factor for the transformation. It is also demonstrated that the changes in measured pH were relatively small, but very large difference in absorption spectra and morphology were observed. Unlike previous work, our synthesis method does not rely upon additional Ag ions, light, and heat during the shape transformation from spherical nanoparticles to triangular nanoprisms. Based on extensive TEM and absorption spectra studies, we proposed a growth mechanism involving the combination of the coalescence and dissolution–recrystallization process, which is a non-point initiated growth mechanism.

## Note

^1^ The reduction potential ($E_{P}^{0}$) of Ag nanoparticles could be calculated by $E_{P}^{0}=E_{\text{bulk}}^{0}-2\gamma V_{\text{M}}/z\text{Fr}$. Here, we use the reduction potential of bulk $E_{\text{bulk}}^{0}$ = 800 mV, the surface tension *γ* = 1.45 J/m^2^, the molar volume *V*_M_ = 10.3 cm^3^/mol, the number of valence electron *z* = 1, and the Faraday's constant *F* = 96,485.34 C/mol. The *r* is the radius of the silver nanoparticles.
